# Motivations for uterus transplantation in women with absolute uterine factor infertility: A systematic review of the literature

**DOI:** 10.1016/j.clinsp.2025.100646

**Published:** 2025-04-13

**Authors:** Luciana Leis, Francisco Tustumi, José Maria Soares-Jr, Edmund Chada Baracat, Luiz Augusto Carneiro-D'Albuquerque, Dani Ejzenberg, Wellington Andraus

**Affiliations:** a Department of Gastroenterology, Hospital das Clínicas, Faculdade de Medicina, Universidade de São Paulo (HCFMUSP), São Paulo, SP, Brazil; b Department of Obstetrics and Gynecology, Hospital das Clínicas, Faculdade de Medicina, Universidade de São Paulo (HCFMUSP), São Paulo, SP, Brazil

**Keywords:** Uterus, Transplantation, Infertility, Motivation

## Abstract

**Introduction:**

Uterine transplantation is indicated for women with absolute uterine factor infertility, which is usually associated with congenital causes (the most common being Mayer-Rokitansky-Kuster-Hauser syndrome) or acquired via hysterectomy. For these women, uterus transplantation is the only possibility for them to be able to conceive and have a biological child. The aim of this study was to understand the motivations that lead women with absolute uterine factor infertility to wish to undergo a uterus transplant.

**Methods:**

A systematic literature review was performed in the following databases: Medline (PubMed), Embase, Cochrane (CENTRAL), Scopus, Web of Science, and LILACS.

**Results:**

439 articles were identified, and nine studies were finally selected. Uterine transplantation was the preferred choice over adoption and surrogacy in most studies (ranging from 45 % to 97.5 %). The main motivations for this type of transplant include the desire to conceive a pregnancy, the desire to have a genetic child, the desire to have control over pregnancy, the physiological repair of the body, the restoration of femininity, and the desire to contribute to science.

**Conclusion:**

The motivations for uterus transplantation go far beyond the desire to get pregnant and include personal and unique motivations.

## Highlights

• Uterine transplantation is the preferred choice for most women with absolute uterine factor infertility.
• The motivations for transplantation go beyond the desire to conceive one's own child.
• Having a genetic child, controlling pregnancy, and restoring femininity are also among the reasons for seeking uterus transplantation.

## Introduction

Nowadays, organ transplants are not just limited to saving lives. In recent years, advances in science have made other types of transplants possible, aiming to improve people's quality of life, such as face, body, and larynx transplants. Uterus Transplantation (UTx) is in this category, and in addition to promoting a better quality of life, it can also provide the propagation of life. UTx is the first temporary transplant. Once the desired number of children is born, the uterus is removed so that the patient does not need to take immunosuppressants for the rest of their life, avoiding long-term side effects.^1^

The UTx is indicated for women with an absolute uterine factor of infertility, such as congenital or acquired causes. Among congenital causes, the most common is Mayer-Rokitansky-Kuster-Hauser (MRKH) syndrome.^2^^,^^3^ Acquired causes include hysterectomy for cervical cancer, postpartum hemorrhage, uterine bleeding, leiomyoma, and others.^2^^,^^4^

It is estimated that one in every 500 women has an absolute uterine factor of infertility, meaning that the only possibility these women have of getting pregnant and giving birth to a biological child is through a UTx.^5^

However, while most transplants involve a single surgery on the receptor, UTx requires a series of procedures: in vitro fertilization, graft implantation, embryo transfer, cesarean section, and graft removal after the conclusion of pregnancy to remove the immunosuppressant. Also, it includes surgery on the donor in cases of living-donors uterus transplantation.^6^

The uterus donation for a transplant can happen in two ways: through the donation of a uterus from a living woman (known or unknown to the receptor) or a deceased woman.^7^^,^^8^

Many patients with absolute uterine factors of infertility will find in the adoption or surrogacy a possible way to build their families. Therefore, it is essential to highlight that a series of personal, financial, legal, and ethical implications may be related to these types of searches, making this path difficult or impossible depending on each country's culture and/or legislation.^9^

It is essential to highlight that infertility often leads to feelings of inferiority and inadequacy. For some women, pregnancy ‒ and the physical changes it brings ‒ serves as tangible proof of their capacity and femininity.^10^ Thus, a uterus transplant would be of great importance for these women.

Given the complexity of the procedures involved in uterine transplantation and the existence of less risky alternatives for motherhood, the question arises about the motivations that lead patients to choose this option. Thus, this study aims to understand the reasons that lead women to choose this therapeutic approach to fulfill their desire to have children.

## Methods

This research followed the PRISMA precepts. The research protocol was submitted for registration in the PROSPERO database (International Prospective Register of Systematic Reviews; http://www.crd.york.ac.uk/PROSPERO), registered under number CRD42024539518.

### Search

The evidence search was performed using Medline (PubMed), Embase, Cochrane (CENTRAL), Scopus, Web of Science, and LILACS databases to carry out the systematic literature review. The articles were manually selected according to the defined eligibility criteria. Two independent authors performed the literature screening. Any disagreement about the inclusion of the final study was solved by discussion. The search was conducted in January 2025.

The following descriptors were used: ("uterus transplant*" OR “womb transplant*”) AND (motivation* OR intention* OR interest* OR perspective* OR expectation* OR psychology) AND (female OR women) AND (infertility OR sterility).

### Inclusion and exclusion criteria

This review included studies that directly or indirectly investigated the motivations for uterus transplantation in women who were potential candidates for it, generally women with congenital absence of the uterus (MRKH syndrome) or women who underwent hysterectomy due to benign or malignant disease or complications during childbirth. Patients who underwent uterus transplantation were also included in the sample. Transgender women, letters, editorials, and guidelines were excluded from this study.

## Results

### Selection of papers

**Fig. 1:**
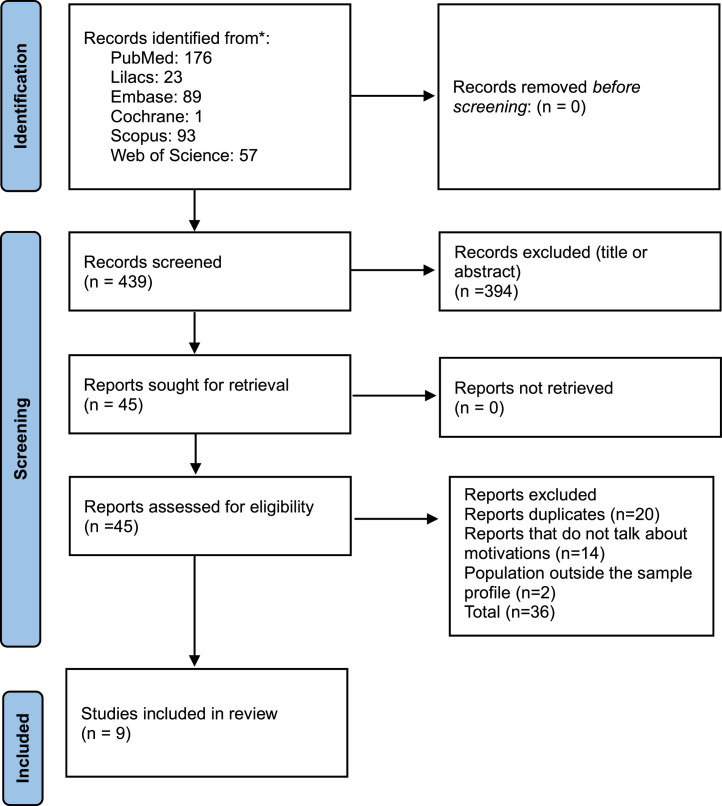
PRISMA flow diagram.

After applying the search strategy, 439 articles were identified in the databases, and eventually, eight articles and one conference abstract were selected^3^^,^^6^^,^^9^^,^^11^, ^12^, ^13^, ^14^, ^15^, ^16^ (Fig. 1).

### Baseline characteristics of the included studies

Sample sizes vary significantly, ranging from 19 to 281 participants. Among the selected studies, with a pooled number of 815 women, the vast majority had MRKH syndrome (716), while the others evaluated patients with acquired absolute uterine factor infertility. The average age reported varied between 26 and 32 years. Three studies were conducted in the USA, two in England, two in France, one in Malaysia, and one in Australia. The baseline characteristics of the included studies are summarized in Table 1.

**Table 1:** Baseline characteristics of the included studies.

Authors (year)	Location	Sample number	Marital status		Pacients profile		Utx	Average age (years old)	Age range (years old)	Methodology
			Single/ Separated	Married/ Stable relationship	MRKH	Acquired uterine factor				
Wall et al. (2021)	Dallas, USA	20	Not informed	Not informed	18	2	Yes	31.9	26‒38	Semi-structured interview
Saso et al. (2016)	London, UK	40	3	37	20^a^	14^a^	No	29	19‒55	Semi-structured interview and questionnaire before and after explanatory material about UTx
Sousa et al. (2023)	France	148	31	116	148	0	No	Not informed	< 25 years: *n* = 50; 25–42 years: *n* = 92; > 42 years: *n* = 6	Questionnaire before and after explanatory material about UTx
Jones et al. (2023)	London, UK	210	15	195	143	67	No	Not informed	20–59	Online questionnaire
Azhary et al. (2023)	Malaysia	59	31	28	59	0	No	29.1	Not informed	Questionnaire after explanatory material about UTx
Pittman et al. (2020)	Australia	57	11	29	47	10	No	32	18‒54	Questionnaire and brief teaching material about UTx.
Fischer et al. (2021)	USA	281	102	81	281		No	28.2	Not informed	Questionnaire before and after followed of educational material
Richards et al. (2019)	USA	19	1	18	11	8	No	30.4	Not informed	Semi-structured interview and FertiQol.
Gauthier et al. (2015)	France	60	20	38	51^a^	7^a^	No	26	18–53	Educational material and questionnaire

UTx, Uterine Transplantation.

a The article does not specify the diagnostic condition of all patients.

Most of the women were married or in a stable relationship. The history of biological children is inconsistently reported, with some studies specifying numbers while others do not define the origin of previous children. Preferences for donor type also show variability. Due to the high clinical heterogeneity among the included studies, it was impossible to perform a quantitative synthesis, and consequently, the results were expressed qualitatively. Table 2 summarizes the history of biological children and individual preferences for uterus transplants.

**Table 2:** History of biological children, and individual preferences for uterus transplant and type of transplant.

Authors/year	Sample number	History of biological children				Preference for transplant	Preference for donor type			
		Biological and gestational	Gestational surrogacy	Adoptive	Stepchildren		Alive and known	Alive and unknown	Deceased	No preference
Wall et al. (2021)	20	0	1	3	1	Not informed	1	17	2	
Saso et al. (2016)	40	3	3	1	0	39 (97.5 %)	Not informed			
Sousa et al. (2023)	148	0	5	4	0	90 (60.8 %)	Not informed			
Jones et al. (2023)	210	0	0	0	0	107 (50.9 %)	Not informed			
Azhary et al. (2023)	59	Not informed				Not informed	6^a^	9^a^	7^a^	32^a^
Pittman et al. (2020)	57	9 Women had children but do not define their origin				27 (47.3 %)	15 (study does not define if the donor is known or unknown)		6	36
Fischer et al. (2021)	281	23 had children but do not define the origin				129 (45.9 %)	47^a^	69^a^	49^a^	
Richards et al. (2019)	19	4	1	1	1	Not informed	There was no consensus on the preferred type of donor			
Gauthier et al. (2015)	60	Not informed				Not informed	Not informed			

a Not all patients answered this question. ^⁎⁎^NE, Not Evaluated.

The majority of studies in this systematic review involved women considered for uterine transplantation. The sole exception was a study by Wall et al.,^11^ which focused on individuals who had already undergone the procedure, regardless of the surgery's outcome.

All studies used questionnaires or interviews to investigate the topic of interest. In four studies, a closed structured questionnaire was used, in which patients had to choose the alternative that most identified with them in the answer, and five studies used semi-directed interviews, allowing for greater exploration of the responses regarding the topics addressed. Only one study used another instrument in its methodology besides the questionnaire/interview: the Fertility and Quality of Life Tool, Fertiqol. This research, carried out by Richards et al.^9^ with 19 patients, used semi-directed interviews to deepen the qualitative data. Although they used Fertiqol to complement the study, it was noteworthy that they did not address the results.

### Knowledge about uterus transplantation

Six of the nine studies selected used educational material explaining uterus transplantation to patients before submitting them to the questions to be investigated.

An Australian survey of 57 potential female UTx candidates found that all participants had varying degrees of knowledge about it. After reading material containing a brief general explanation about the UTx procedure, 60.9 % of the cohort reported that they would still consider it to achieve motherhood, regardless of the potential risks mentioned.^6^

Saso et al.^12^ conducted a study that evaluated the knowledge about UTx in 40 women interested in undergoing this type of surgery. They used an educational video about UTx (evaluating knowledge about transplantation before and after the video). They found that there was statistical significance (*p* < 0.01) for increased understanding of the risks and benefits associated with transplantation after the video, where patients (95 %) believed that the benefits outweighed the risks.

As in the two studies cited above, the relativization of risks in favor of UTx was a finding observed in most of the studies considered here, such as that of Wall et al.,^11^ Fischer et al.,^13^ Gauthier et al.^14^ and Richards et al.^9^

### Preference for donor type for uterus transplantation

Regarding the preference for the type of uterus donor, five studies addressed this issue with patients, and 32 % of the women (*n* = 296) who answered this inquiry objectively preferred a living and unknown donor, 22 % had no preference, 21 % preferred a deceased donor, and 18 % preferred a living and known donor. A study conducted by Pittman et al.^6^ found that 26 % of the women in their sample preferred a living donor; it did not specify whether they were known. Although a qualitative survey conducted by Richards et al.^9^ addressed the preference for the type of donor with the participating women, it did not conclude the subject.

### Preference for uterus transplantation over other means of access to maternity

Uterus transplantation as a preferred choice over adoption and surrogacy was addressed in five of the included studies. In almost all, this transplantation type was the preferred choice. In a study conducted by Saso et al.^12^ with 40 women, the preference reached 97.5 %, while in the other studies, this preference varied from 45.9 to 60.8 %.

Among the population studied, it is important to highlight those 60 women who already had a child via adoption, surrogacy, or pregnancies before losing their uterus or stepchildren (two of the studies did not mention this information).^14^^,^^15^

### Motivations for UTx

Among the main motivations that lead women to want to undergo this type of surgery, the desire to gestate their own child was the main reason in most studies (8 of the nine studies included in this article). The desire to have a genetic child also proved to be of great importance for a large part of the population studied (Table 3).

**Table 3:** Main motivations for desiring for uterus transplantation. Patients could answer more than one response option.

	Motivations for uterus transplantation							
Authors (year)	Desire to conceive the own baby	Desire to have a genetic child	Desire to have control of pregnancy	Restoration of feminity	Complete the family	Repair of the physiology of the body	Restoration of the autonomy of the body	Desire to contribute with the science
Wall et al. (2021)	Only present qualitative data							
Saso et al. (2016)		90 %	90 %		8 %			
Sousa et al. (2023)	89 %	60 %						
Jones et al. (2023)	53 %	37 %						
Azhary et al. (2023)	58 %							
Pittman et al. (2020)	46 %							
Fischer et al. (2021)	80 %							
Richards et al. (2019)	Only present qualitative data							
Gauthier et al. (2015)	Only present qualitative data							

In addition to the desire to conceive and have a genetic child, a uterus transplant would also be a way for these women to have greater control over their pregnancy, as they would not need to involve a third person in this process (as occurs with surrogacy). The transplant also represents a possibility of physiological repair of the body and restoration of femininity through the newly implanted organ. Furthermore, three studies indicated the desire to contribute to science as one of the relevant reasons for choosing a transplant.

## Discussion

Uterus transplantation, although it can make many women's dreams of gestational and biological motherhood come true, involves risks for both mother and baby. These risks seem to be underseen by women who wish to undergo this type of transplant due to the inconsolable need to become mothers.^3^^,^^6^^,^^12^^,^^13^^,^^16^

The studies by Saso et al.^12^ and Pittman et al.^6^ demonstrated the patients’ tendency to minimize risks. Even after obtaining objective information about UTx and its possible complications, patients assessed that the benefits outweighed the risks, such as the motivation and, in a certain way, idealization for this surgery.

Wall et al.,^11^ in a qualitative study of women who underwent UTx, found that many of them described their experiences with UTx as different from what they expected, such as being easier, more complex, more intense, or different in terms of treatment schedule. The research also found that the trust that these women placed in the team that would perform the transplant was an important psychological strategy used to alleviate concerns related to the potential risks that this type of surgery entails.

The personal motivations involved in the desire for pregnancy, combined with the psychological strategies used to face possible fears involved in this type of surgery, allow the vast majority of women in the studies consulted here to place uterus transplantation as their preferred choice as a way of fulfilling their desire for motherhood, concerning adoption or surrogacy.

Regarding the preference for the type of uterus donor, among the patients who expressed their position on this aspect (296), 50 % preferred a living donor (known or unknown), and 21 % preferred a deceased donor. Sanner,^17^ in a study with organ recipients, found that there are emotional differences related to the fact that the donor is alive or deceased, in which the psychological reactions associated with the deceased donor seemed to require greater emotional work from the patients, especially considering that many had fantasies of being able to have changes in themselves influenced by the type of personality of the donor. This type of fantasy was not verified when the donors were alive and known, as it seems to be easier to delimit one's space with an identified and concrete person than with an anonymous deceased donor, in which the field is open to fantasy.

In cases where a preference is given to a living and unknown uterus donor, even though there is also room for fantasies to be projected since the donor is anonymous, it is still different from the patient receiving an organ from a deceased anonymous donor since receiving an organ from a dead person in one's body is usually not an easy emotional experience. Patients who have received organs from deceased donors speak of conflicting feelings between wanting to live and, at the same time, someone having to die for this to happen.^17^, ^18^, ^19^

An interesting fact to be highlighted in this systematic literature review is that 60 (7 %) women included in this review, who were already mothers through adoption, surrogacy or who had stepchildren, still wanted to undergo a UTx, demonstrating that the reasons for undergoing this type of transplant go far beyond the way to motherhood and that the desire to experience a pregnancy and/or the possibility of having a genetically related child seems to be something of great value to these women.

Among the main motivations for UTx, the authors found that the desire to carry one's own child led to most of the studies included here. In qualitative studies, this desire transcends the experience of simply producing a baby but also concerns the possibility of relating and bonding with the child long before birth and seeing the physical transformations of pregnancy in one's body.^9^^,^^12^

Being pregnant is a feminine capacity, and the inability to carry a child inevitably affects women's self-esteem. Many of them describe feeling “incomplete”, referring not only to the sadness of not being able to be pregnant but also to the loss of their reproductive organs as a vital center of creativity.^20^ Lanius^10^ emphasizes that for some women, only gestation, through the real mark of pregnancy on their bodies, would be able to prove to them their capacity and femininity - unlike this recognition through symbolic means.

A study conducted by Jones et al.^21^ with 182 transgender women further explained the connection between the uterus and femininity. In this study, most patients (94 %) believed that the ability to gestate and menstruate (88 %) would increase their perception of femininity, helping them feel more like a woman.

The motivation for seeking UTx as a means of having a genetic child also emerged among the primary reasons for women wanting to undergo this type of surgery. The chance of having a genetically related child is usually of great significance to most women, and the inability to do so can represent a narcissistic wound.^22^ As early as 1914, Freud^23^ noted that the child serves as a narcissistic extension of the parents because, through the child, it is possible to see reflections of oneself in the beloved, to fulfill old dreams projected onto the child, and to achieve a sense of “immortality”, as a part of oneself may continue to exist after death.

It is also important to emphasize that although surrogacy is a possible means of achieving biological motherhood, not all women can resort to this option. This is especially true considering that not all women have close relatives willing to carry a child for them or have the financial resources to pursue surrogacy, given the high costs involved. Additionally, in some countries, surrogacy is prohibited, which makes access to this option even more challenging as patients may need to travel to other countries where this type of treatment is allowed in order to try to have a child.

The desire for control over pregnancy was one of the motivations for UTx, as identified in two of the studies included in the present research.^9^^,^^12^ Not involving a third party in this process ‒ especially concerning patients considering treatment through surrogacy ‒ and being able to manage this experience without needing to trust someone else to carry their baby is a goal for many women. Additionally, Richards et al.^9^ pointed out that the transplant could provide these patients with greater reproductive autonomy, allowing for privacy during the gestation process, similar to what women with a uterus experience.

The qualitative research by Gauthier et al.^14^ brought important contributions that complement the understanding of the reasons that lead women to desire a UTx. In addition to the desire to conceive and have a genetic child, these researchers also found that the transplant would be a possibility of restoring femininity and physiological repair of the body through the implantation of the missing uterus. The research by Leis & Modelli^24^ with infertile women confirms these findings since they realized that the majority of patients sought to have a child through assisted reproduction treatments in an attempt to feel more “woman” through motherhood.

For women who lost their uterus during childbirth, after the birth of their child, the transplant would be a possibility for them to complete their families and a way to better deal with the trauma of hysterectomy.^14^

An interesting and unexpected finding that emerged in the systematic review is that many women interested in UTx felt motivated ‒ among other reasons ‒ to undergo this type of surgery due to the desire to contribute to science, given that UTx is still an experimental surgery that requires further studies and improvements to the technique in order to evolve.

Gauthier et al.,^14^ in a survey of potential UTx candidates, highlighted that participation in a research protocol on UTx was seen as an altruistic and militant gesture, which attested to the feeling of belonging and help in the development of research for a community of women who are generally excluded from medical assistance proposals for maternity.

Given the scarcity of studies on the motivations of women with absolute uterine factor infertility who desire a uterus transplant, the authors recommend that future research broaden the findings of this study. Additionally, the authors observed that many of the studies included here prompted women to speak solely about the significance of pregnancy to justify their choice of UTx, as if this were the only reason for this pursuit. The lack of openness in many studies to explore other motivations for seeking this type of transplant may have further exaggerated the significance of pregnancy in these results.

## Conclusion

Uterus transplantation is an innovative technique that has made it possible for many women around the world to fulfill their desire for gestational and biological motherhood. Through this systematic review of the literature on the motivations that lead women with absolute uterine infertility to wish to undergo uterus transplantation, it was found that the reasons for this choice go beyond the simple desire to carry their own child. They include personal and unique motivations that reflect the symbolic importance of the uterus in each woman's individual trajectory.

## Declarations

Ethics: The local ethics committee approved the study.

Data availability statement: The datasets generated during and/or analyzed during the current study are available from the corresponding author upon reasonable request.

Abbreviations: UTx, Uterus Transplantation; MRKH, Mayer-Rokitansky-Küster Hauser syndrome.

## Conflicts of interest

The authors declare no conflicts of interest.
